# The causal relationship between sleep characteristics and multi-site pain perception: a two-sample Mendelian randomization study

**DOI:** 10.3389/fnins.2024.1428951

**Published:** 2024-08-13

**Authors:** Yulai Yin, Xiaoyu Zhang

**Affiliations:** ^1^Cangzhou Central Hospital, Hebei Medical University, Cangzhou, China; ^2^Department of Thyroid and Breast Surgery III, Cangzhou Central Hospital, Cangzhou, China

**Keywords:** sleep traits, pain, Mendelian randomization, instrumental variables, single-nucleotide polymorphisms

## Abstract

**Objective:**

This Mendelian Randomization (MR) study aims to explore the potential causal relationships between four sleep traits and pain in 10 different body sites.

**Materials and methods:**

The study utilizes exposure and outcome data from the GWAS database, employing the Inverse Variance Weighting Method (IVW) for primary causal estimates. Cochran Q and Rücker Q heterogeneity tests are conducted using IVW and MR-Egger methods, with the Egger-intercept method for pleiotropy testing, leave-one-out sensitivity analysis, and calculation of F-statistics to assess the presence of weak instrument bias.

**Results:**

The study reveals that genetically predicted insomnia significantly increases the risk of unspecified pain, chest pain, gum pain, upper abdominal pain, and lower abdominal pain occurrence. Daytime napping is associated with a moderate reduction in the likelihood of joint pain but may concomitantly elevate the risk of chest pain, upper abdominal pain, and generalized abdominal pain. Neither sleep chronotype nor sleep duration demonstrated a definitive causal relationship with pain perception.

**Conclusion:**

This study elucidates the causal relationships between four sleep characteristics and pain across 10 different body regions. Overall, the contribution of insomnia and sleep deficiency to pain in multiple body regions is more pronounced. Conversely, the association between adequate sleep and the likelihood of somatic pain is relatively lower and less significant.

## 1 Introduction

Sleep (Albqoor and Shaheen, 2021; Mason et al., 2021; Chunnan et al., 2022) is a fundamental human need, essential for maintaining vitality and engaging actively in social life. However, global sleep trends in 2023 highlight significant regional disparities in sleep patterns, behaviors, and challenges faced. Research from the Yong Loo Lin School of Medicine at the National University of Singapore, involving over 220,000 participants, suggests that Asians experience less sleep compared to other global populations. Furthermore, the 2023 Global Sleep Survey by ResMed indicates that 80% of adults suffer from sleep disruptions related to sleep quality (Khan and Aouad, 2022; Papatriantafyllou et al., 2022). Short-term sleep deprivation can lead to irritability, difficulty concentrating during the day, and excessive daytime sleepiness, while long-term deprivation increases the risk of cardiovascular and cerebrovascular diseases, diabetes, obesity, depression, and cognitive dysfunction, thereby endangering health. Thus, regulating sleep and ensuring adequate rest is crucial for health.

Pain is an unpleasant emotional experience associated with tissue damage or described as originating from a bodily sensation (Wang and Mullally, 2020; Cheng et al., 2021). It is a complex and subjective response to adverse stimuli, involving sensory, emotional, cognitive, and social factors. The perception of pain is linked to various brain regions responsible for processing pain signals, emotional areas, and cognitive functions that affect pain perception. Acute pain, a direct response to injury or disease, serves a protective function, typically diminishing as tissues heal or stimuli are removed. Chronic pain, however, persists for months or years and can continue even after the initial injury has healed. Chronic pain not only affects the physical body but also has profound impacts on emotional and psychological well-being, leading to anxiety and depression (Brammer and Miller, 2022; Yao et al., 2023). Addressing chronic pain and its causes promptly is vital for overall health.

Currently, sleep disorders and pain impose significant psychological and physiological burdens on individuals. Moreover, they place substantial pressure on societal and healthcare resources (Chang et al., 2022). Large-scale observational studies have found that 67 to 88% of individuals with chronic pain disorders experience sleep disturbances, and at least 50% of those with insomnia, the most common sleep disorder, suffer from chronic pain (Finan et al., 2013). Consequently, researchers in the fields of sleep and pain have begun to investigate the relationship between the two. Notably, four longitudinal studies (Affleck et al., 1996; Stone et al., 1997; Drewes et al., 2000; Raymond et al., 2001) have observed directional effects of sleep problems on pain, while five longitudinal studies (Nicassio and Wallston, 1992; Affleck et al., 1996; Drewes et al., 2000; Raymond et al., 2001; Riley et al., 2001) have noted directional effects of pain on sleep problems. Regrettably, current research primarily focuses on the correlation between sleep and pain, lacking exploration of their causal relationship. Additionally, most studies concentrate on perioperative patients, where pain perception might be influenced by surgical incisions or other factors, introducing bias. Moreover, research on sleep and pain tends to categorize pain based on its location, with scant attention given to the four distinct sleep characteristics: sleep duration, insomnia, chronotype, and daytime napping. Most studies narrowly focus on insomnia as the sole exposure. In summary, exploratory studies on the causal relationship between sleep characteristics and pain perception are sparse, often limited to specific pain sites, and hence lack generalizability. Traditional observational studies struggle to avoid confounding factors such as mood, external environment, and social influences. Investigating the causal relationship between sleep and pain will elucidate the mechanisms linking the two, potentially enhancing pain management in postoperative and late-stage cancer patients through interventions targeting molecular receptors or improving sleep conditions. This research holds significant potential for improving patient outcomes and reducing the global burden of disease. Furthermore, the detailed exploration of this causal relationship will offer valuable contributions to the health of clinical and subclinical populations, providing individualized health guidance based on scientific evidence for diverse sleep characteristics across different age groups in society.

Mendelian randomization (MR; Carter et al., 2021; Birney, 2022; Bonilla et al., 2023), a research method based on genome-wide association study (GWAS) databases, offers evidence akin to randomized controlled trials (RCTs). Utilizing single nucleotide polymorphisms (SNPs) associated with exposure factors as instrumental variables, MR explores causal relationships with disease outcomes. MR studies are less prone to confounding factors, simple to conduct, time-efficient, and free from ethical concerns, allowing for the investigation of reverse causality (Meisinger and Freuer, 2022; Lu and Ma, 2023). With appropriate instrumental variables and after excluding confounders, MR provides strong causal evidence, supporting early clinical screening and interventions.

This study employs two-sample Mendelian randomization to investigate the causal associations between four sleep traits (sleep duration, insomnia, chronotype, and daytime napping) and 10 outcomes related to pain (limb, back, neck, head, abdominal, eye, chest, precordial, upper and lower abdominal, gum, joint, and back pain). Based on the findings, this research offers insights for early clinical interventions.

## 2 Materials and methods

### 2.1 Data sources

The data related to exposures and outcomes used in this study were derived from the GWAS database.^1^ Given the public accessibility of the data, ethical committee review was not required for this study. The original research involving the study data has received ethical committee approval. Detailed information on the exposure and outcome data can be found in Table 1. Sleep duration was analyzed as a continuous variable, which was then categorized into two distinct groups. A sleep duration of less than 7 h was classified as short sleep duration, whereas a duration of 9 h or more was classified as long sleep duration. Sleep durations between 7 and 9 h were deemed normal. Extreme values, such as sleep durations of less than 3 h or more than 18 h, were excluded from the analysis. Insomnia, a prevalent sleep disorder, is characterized by difficulty initiating or maintaining sleep, resulting in daytime impairment despite sufficient opportunity for sleep. Chronotype refers to an individual’s preference for early or late sleep times, also known as circadian preference. A daytime nap is defined as a short period of sleep or rest during the day, typically lasting from a few minutes to up to an hour, aimed at restoring energy, enhancing alertness, and improving overall mood. Pain in 10 different body locations was defined as subjective pain perception in the corresponding anatomical regions.

**Table 1:** Data characteristics and sources.

Exposure/Outcome	Traits	ID	Population	Websites
Exposure	Sleep duration	ukb-a-9	European	https://gwas.mrcieu.ac.uk/datasets/?trait__icontains/ukb-a-9
Exposure	Sleeplessness / insomnia	ukb-b-3957	European	https://gwas.mrcieu.ac.uk/datasets/ukb-b-3957/
Exposure	Chronotype	ieu-b-4862	European	https://gwas.mrcieu.ac.uk/datasets/ieu-b-4862/
Exposure	Daytime nap	ebi-a-GCST011494	European	https://gwas.mrcieu.ac.uk/datasets/ebi-a-GCST011494/
Outcome	Pain (limb, back, neck, head abdominally)	finn-b-PAIN	European	https://gwas.mrcieu.ac.uk/datasets/finn-b-PAIN/
Outcome	Ocular pain	finn-b-H7_OCUPAIN	European	https://gwas.mrcieu.ac.uk/datasets/finn-b-H7_OCUPAIN/
Outcome	Chest pain	ukb-b-10771	European	https://gwas.mrcieu.ac.uk/datasets/ukb-b-10771/
Outcome	Precordial pain	ukb-b-20386	European	https://gwas.mrcieu.ac.uk/datasets/ukb-b-20386/
Outcome	Abdominal pain	ukb-b-6223	European	https://gwas.mrcieu.ac.uk/datasets/ukb-b-6223/
Outcome	Pain localized to upper abdomen	ukb-b-6608	European	https://gwas.mrcieu.ac.uk/datasets/ukb-b-6608/
Outcome	Pain localized to lower abdomen	ukb-b-17456	European	https://gwas.mrcieu.ac.uk/datasets/ukb-b-17456/
Outcome	Painful gums	ukb-b-11161	European	https://gwas.mrcieu.ac.uk/datasets/ukb-b-11161/
Outcome	Pain in joint	ukb-b-13019	European	https://gwas.mrcieu.ac.uk/datasets/ukb-b-13019/
Outcome	Back pain	ebi-a-GCST90018797	European	https://gwas.mrcieu.ac.uk/datasets/ebi-a-GCST90018797/

### 2.2 Selection of instrumental variables

**Figure 1:**
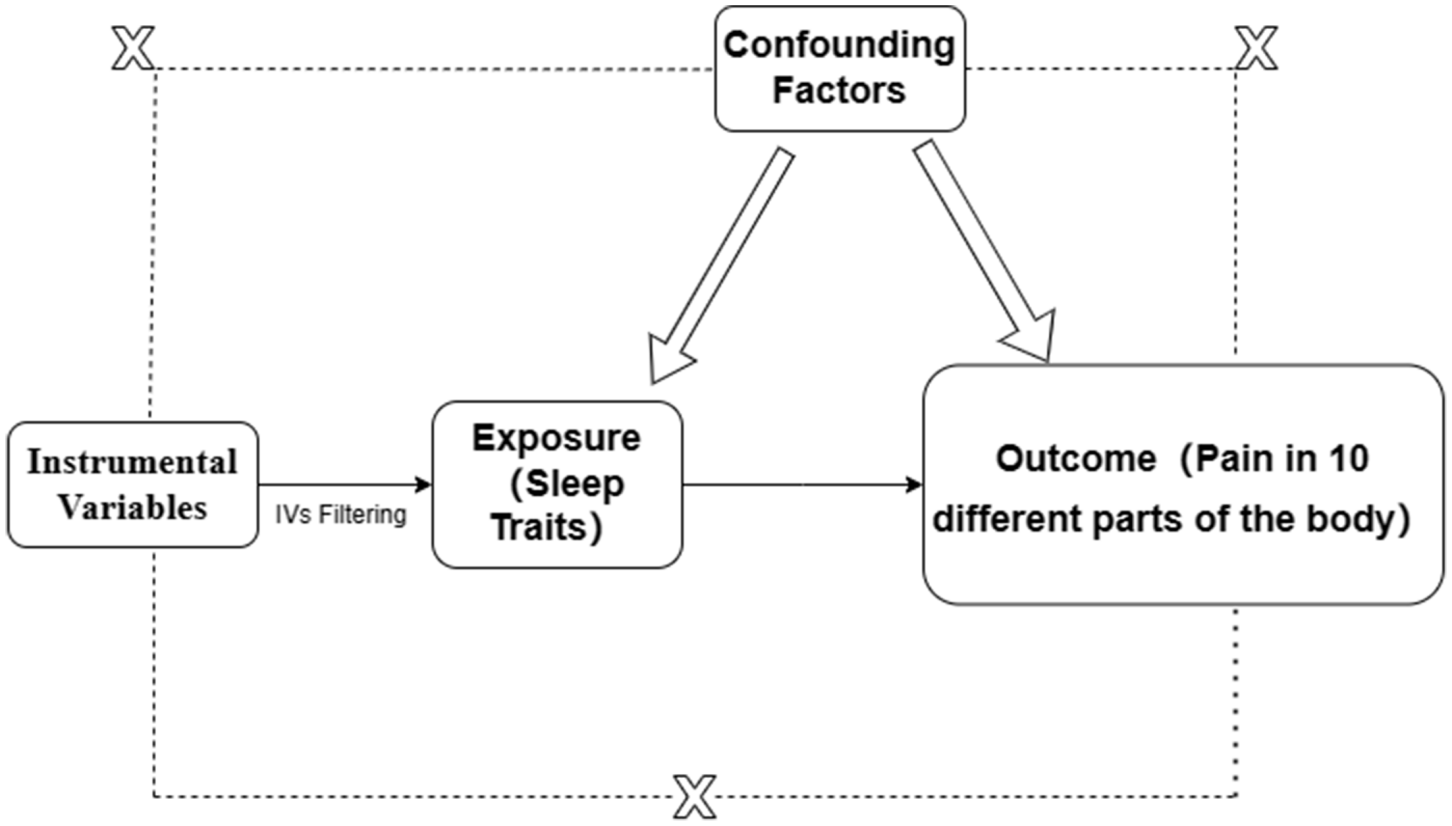
Principle of instrumental variables selection.

Single nucleotide polymorphisms (SNPs) are selected as instrumental variables (IVs) must meet the following criteria: (1) The IV should influence the outcome solely through the exposure; (2) The IV must not affect the outcome through any confounders; (3) The IV should not have a direct effect on the outcome. The specific principle is illustrated in Figure 1.

Initially, SNP data for four sleep characteristics—sleep duration, insomnia, chronotype, and daytime napping (Sang et al., 2022; Yang et al., 2023)—were extracted online from the GWAS database. SNPs significantly associated with these four exposure factors were selected using a threshold of *p* < 5 × 10^−8. To mitigate the effects of linkage disequilibrium (LD), SNPs with an r^2 < 0.001 and within 10,000 kb distance were excluded to eliminate those with weak correlation or proximity that could lead to LD. Furthermore, the F-statistic, F = R^2·(n-k-1)/[k·(1-R^2)], was calculated to exclude weak instrumental variables, where R^2 is the correlation between the instrumental variable and the exposure, and *F* > 10 was used as the selection threshold. Subsequently, SNPs associated with 10 outcomes related to pain—including limb, back, neck, head, abdominal, eye pain, chest pain, precordial pain, full abdominal pain, upper and lower abdominal pain, gum pain, joint pain, and back pain—were extracted based on the selected exposure-related instrumental variables. The exposure and outcome data were then harmonized, and palindromic SNPs were removed. Finally, a two-sample Mendelian randomization analysis was conducted.

### 2.3 MR analysis

The current study primarily employed the Inverse Variance Weighted (IVW) method, along with MR-Egger, Weighted Median, Simple Mode, and Weighted Mode methods for MR analysis. The IVW method was the main approach to investigate the causal relationships between four sleep characteristics and pain in 10 different body parts. A *p*-value <0.05 indicated a statistically significant causal association between exposure and outcome.

### 2.4 Sensitivity analysis

#### 2.4.1 Heterogeneity test

Cochran Q and Rücker Q heterogeneity tests were conducted using the IVW and MR-Egger methods. Significant heterogeneity indicated by the Cochran Q test may suggest variability among instrumental variables or direct associations between some IVs and the outcome variable, indicating potential omitted variable bias. A significant Q statistic necessitates cautious interpretation of results and might require correction for heterogeneity using alternative MR methods. The Rücker Q test, an advanced method for global heterogeneity assessment, is particularly suitable when multiple IVs are involved. A *p*-value >0.05 indicates no significant heterogeneity. In the presence of heterogeneity, the MR PRESSO method was used to remove outliers.

#### 2.4.2 Horizontal pleiotropy test

The Egger-intercept method was utilized for testing horizontal pleiotropy, with a *p*-value >0.05 indicating no evidence of pleiotropy in this study. Additionally, a leave-one-out analysis was conducted to verify result robustness by iteratively removing one SNP at a time and observing the impact of the removed SNP on the outcome. To prevent potential confounding from affecting heterogeneity and pleiotropy, each SNP’s rsid was searched in PhenoScanner^2^ to exclude SNPs associated with traits other than the exposure. Sensitivity analyses were then repeated.

### 2.5 Statistical analysis

Statistical analyses were performed using R software (version 4.3.1). MR analyses were conducted using the “TwoSampleMR” package. Figures were generated using R packages such as “ggplot2,” “dplyr,” “purrr,” and “readr.” Flowcharts were created with the online tool draw.io.^3^ In the statistical analysis results, a *p*-value <0.05 was considered statistically significant.

## 3 Results

### 3.1 Instrumental variable selection

During the selection process of instrumental variables, SNPs associated with four sleep characteristics—sleep duration, insomnia, chronotype, and daytime napping (*p* < 0.05)—were initially extracted from the GWAS database, amounting to 1,685, 2,654, 4,552, and 11,747 SNPs, respectively. Following the exclusion criteria of r^2 < 0.001 and distance ≤10,000 kb to eliminate SNPs with weak correlation or proximity that could lead to linkage disequilibrium, and an *F*-value >10 to exclude weak instrumental variables, 1,642, 2,612, 4,520, and 11,643 SNPs were removed, respectively. Ultimately, 43, 42, 32, and 104 SNPs related to the exposures were selected as instrumental variables. Detailed information on the instrumental variables is available in Supplementary File 1.

### 3.2 MR analysis results

**Figure 2:**
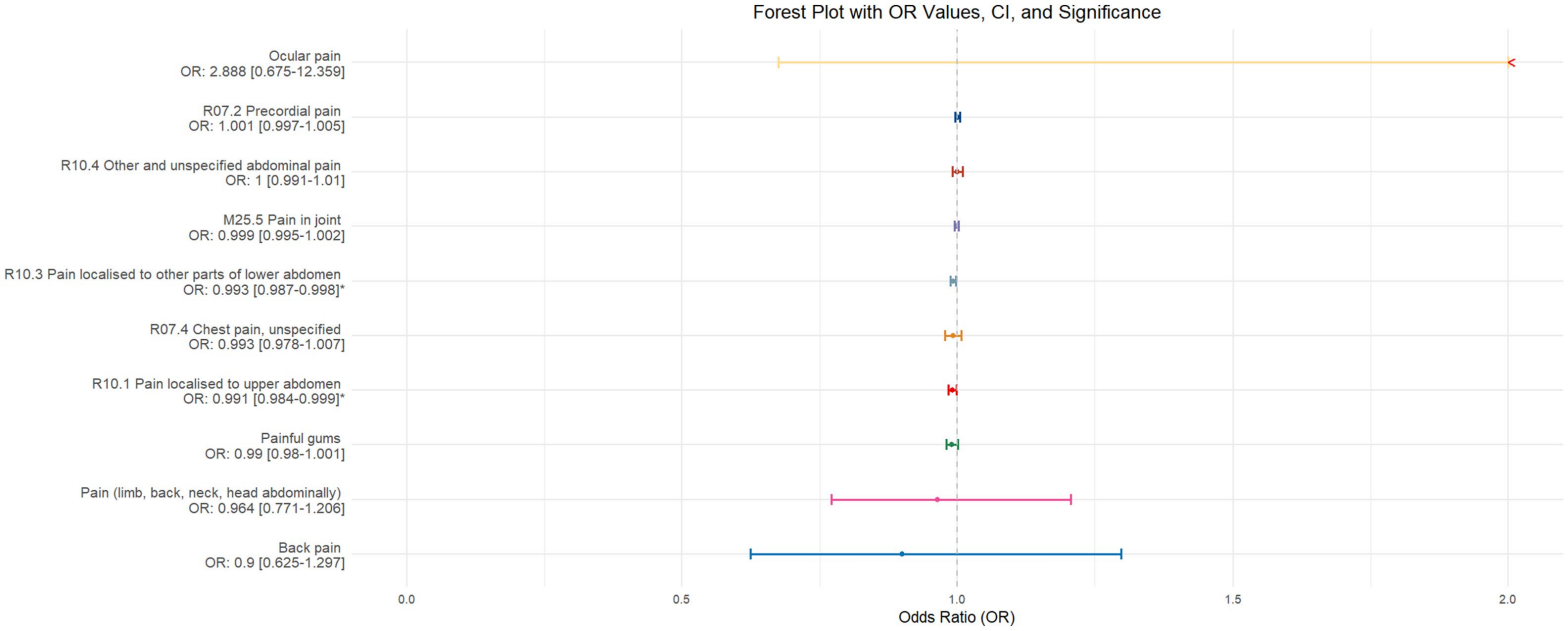
This forest plot is constructed from the MR analysis results using the inverse variance weighted (IVW) method, with sleep duration as the exposure factor and the sensation of pain in 10 different locations as the outcome. The outcomes are arranged in descending order based on the odds ratio (OR) values. An asterisk (*) indicates that the MR analysis results using the IVW method are statistically significant. In this context, a leftward arrow indicates that both the OR value and the upper limit of the OR are not fully displayed, whereas a rightward arrow signifies that only the upper limit of the OR is not fully displayed.

**Figure 3:**
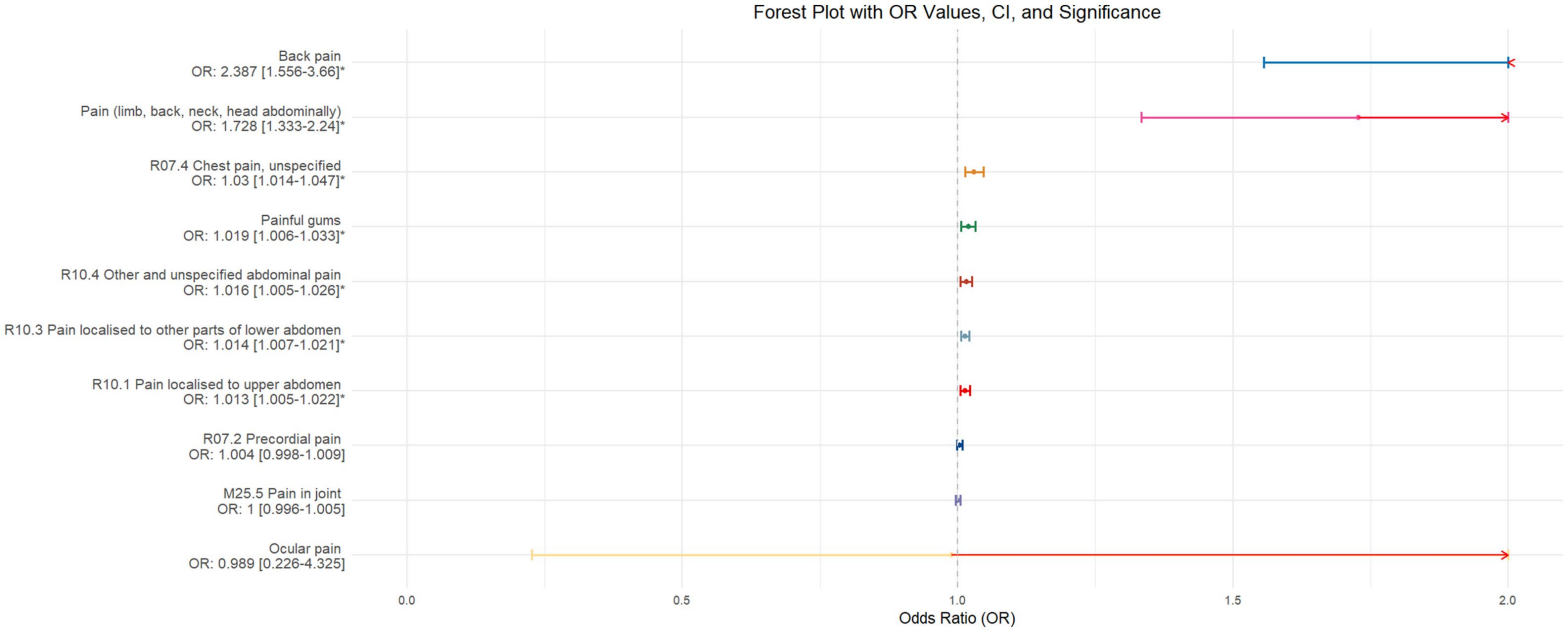
This forest plot is drawn from the MR analysis results using the inverse variance weighted (IVW) method, with insomnia as the exposure factor and the sensation of pain in 10 different locations as the outcome. The outcomes are organized in descending order according to their odds ratio (OR) values. An asterisk (*) signifies that the MR analysis results conducted with the IVW method have statistical significance. In this context, a leftward arrow indicates that both the OR value and the upper limit of the OR are not fully displayed, whereas a rightward arrow signifies that only the upper limit of the OR is not fully displayed.

**Figure 4:**
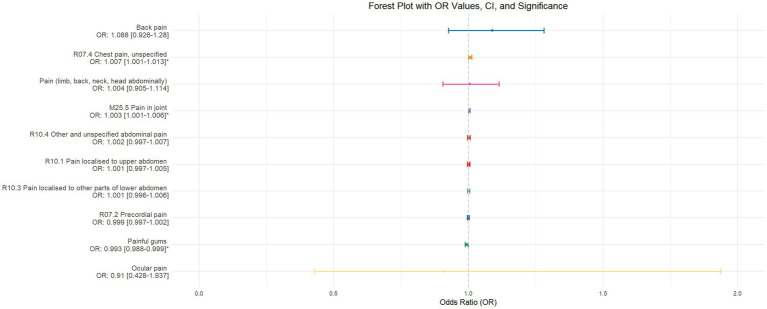
This forest plot is generated from the MR analysis results using the inverse variance weighted (IVW) method, with circadian rhythm as the exposure factor and the sensation of pain in 10 different locations as the outcome. The outcomes are sequenced in descending order based on their odds ratio (OR) values. An asterisk (*) indicates that the MR analysis results obtained through the IVW method are of statistical significance. In this context, a leftward arrow indicates that both the OR value and the upper limit of the OR are not fully displayed, whereas a rightward arrow signifies that only the upper limit of the OR is not fully displayed.

**Figure 5:**
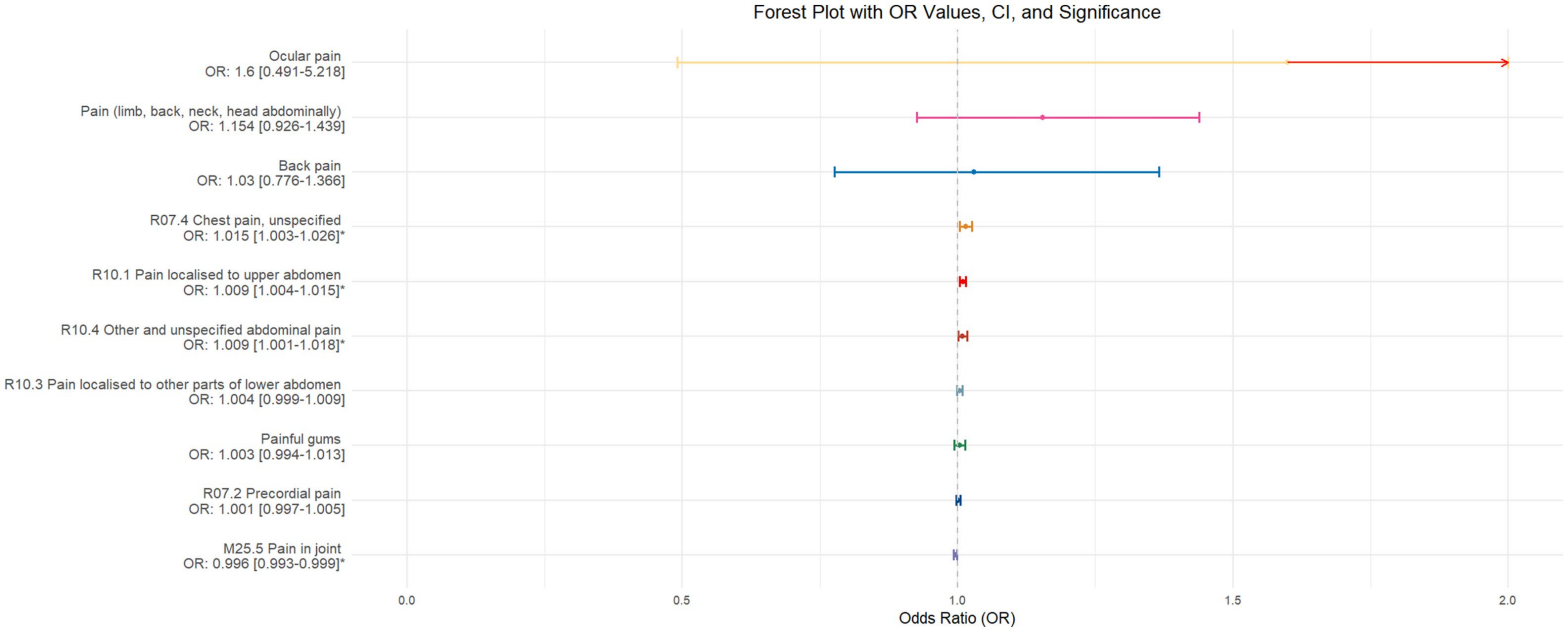
This forest plot is produced from the MR analysis results using the inverse variance weighted (IVW) method, with daytime napping as the exposure factor and the sensation of pain in 10 different locations as the outcome. The outcomes are ordered from top to bottom according to the odds ratio (OR) values. An asterisk (*) denotes that the MR analysis results conducted with the IVW method are statistically significant. In this context, a leftward arrow indicates that both the OR value and the upper limit of the OR are not fully displayed, whereas a rightward arrow signifies that only the upper limit of the OR is not fully displayed.

Using inverse-variance weighted (IVW) as the primary analytical method for Mendelian randomization (MR), our analysis reveals that with increasing sleep duration, the probability of experiencing lower abdominal pain and upper abdominal pain significantly decreases (*p* < 0.05), with odds ratios (OR) [95% confidence intervals (CI)] of 0.993 [0.987–0.998] and 0.991 [0.984–0.999], respectively, as illustrated in Figure 2. Insomnia was associated with an increased probability of back pain, pain (including limb, back, neck, head, abdominal), chest pain, gum pain, full abdominal pain, lower abdominal pain, and upper abdominal pain (*p* < 0.05), with OR[95%CI] of 2.387[1.556–3.660], 1.728[1.333–2.240], 1.030[1.014–1.047], 1.019[1.006–1.033], 1.016[1.005–1.026], 1.014[1.007–1.021], and 1.013[1.005–1.022], respectively; detailed results are shown in Figure 3. A causal relationship was found between chronotype (Montaruli et al., 2021; Yuan et al., 2023) and chest pain, joint pain, and gum pain (*p* < 0.05), with “night owls” more likely to experience chest and joint pain, and “morning larks” more likely to suffer from gum pain, with OR [95%CI] of 1.007[1.001–1.013], 1.003[1.001–1.006], and 0.993[0.988–0.999], respectively; detailed results are depicted in Figure 4. Daytime napping showed a statistically significant causal relationship with chest pain, upper abdominal pain, full abdominal pain, and joint pain (*p* < 0.05), suggesting that napping might increase the risk of chest, upper abdominal, and full abdominal pain, while possibly reducing the risk of joint pain, with OR[95%CI] of 1.015[1.003–1.026], 1.009[1.004–1.015], 1.009[1.001–1.018], and 0.996[0.993–0.999], respectively; detailed results are presented in Figure 5.

### 3.3 Sensitivity analysis

Sensitivity analyses were conducted using the inverse variance weighted (IVW) method and MR-Egger method for Cochran Q and Rücker Q heterogeneity tests. A *p*-value greater than 0.05 suggests the presence of heterogeneity. In cases of heterogeneity, the MR-PRESSO method was utilized, selecting 1,000 distributions to remove outliers. If the Egger-intercept test for horizontal pleiotropy was performed and resulted in a *p*-value greater than 0.05, it indicates that there is no horizontal pleiotropy in this study. Results of heterogeneity tests and horizontal pleiotropy tests prior to outlier removal can be found in Supplementary File 2.

### 3.4 Validation

Given that some exposure and outcome factors are derived from the same database, there is a potential risk of sample overlap biasing our study results (Burgess et al., 2016). To fundamentally mitigate this bias and prevent the inflation of Type I errors, we have adjusted the data sources for some exposure and outcome variables in the validation phase. This adjustment aims to minimize the likelihood of sample overlap as much as possible. The specific changes in data sources are detailed in Table 2.

**Table 2:** Updated data characteristics and sources for validation MR analysis (unmentioned outcome and exposure factors remain unchanged).

Exposure/Outcome	Traits	Original ID	New ID	Population
Exposure	Sleep duration	ukb-a-9	ieu-a-1088	European
Exposure	Sleeplessness / insomnia	ukb-b-3957	ukb-a-13	European
Exposure	Chronotype	ieu-b-4862	ebi-a-GCST003837	European
Outcome	Back pain	ebi-a-GCST90018797	ukb-b-11241	European

**Figure 6:**
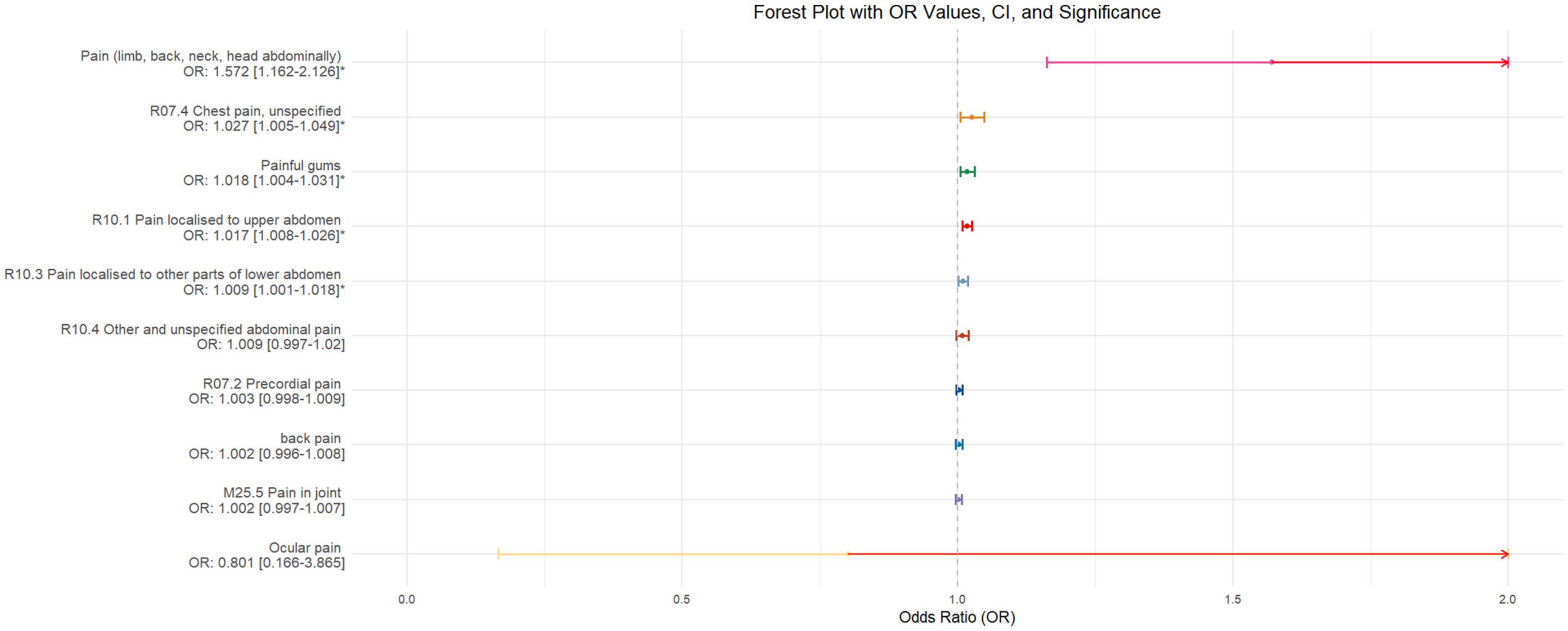
Forest plot of IVW MR analysis results using insomnia as the exposure factor and pain perception in 10 different body regions as outcomes, ordered by OR value from top to bottom. * Indicates statistically significant MR analysis results by IVW method. In this context, a leftward arrow indicates that both the OR value and the upper limit of the OR are not fully displayed, whereas a rightward arrow signifies that only the upper limit of the OR is not fully displayed.

**Figure 7:**
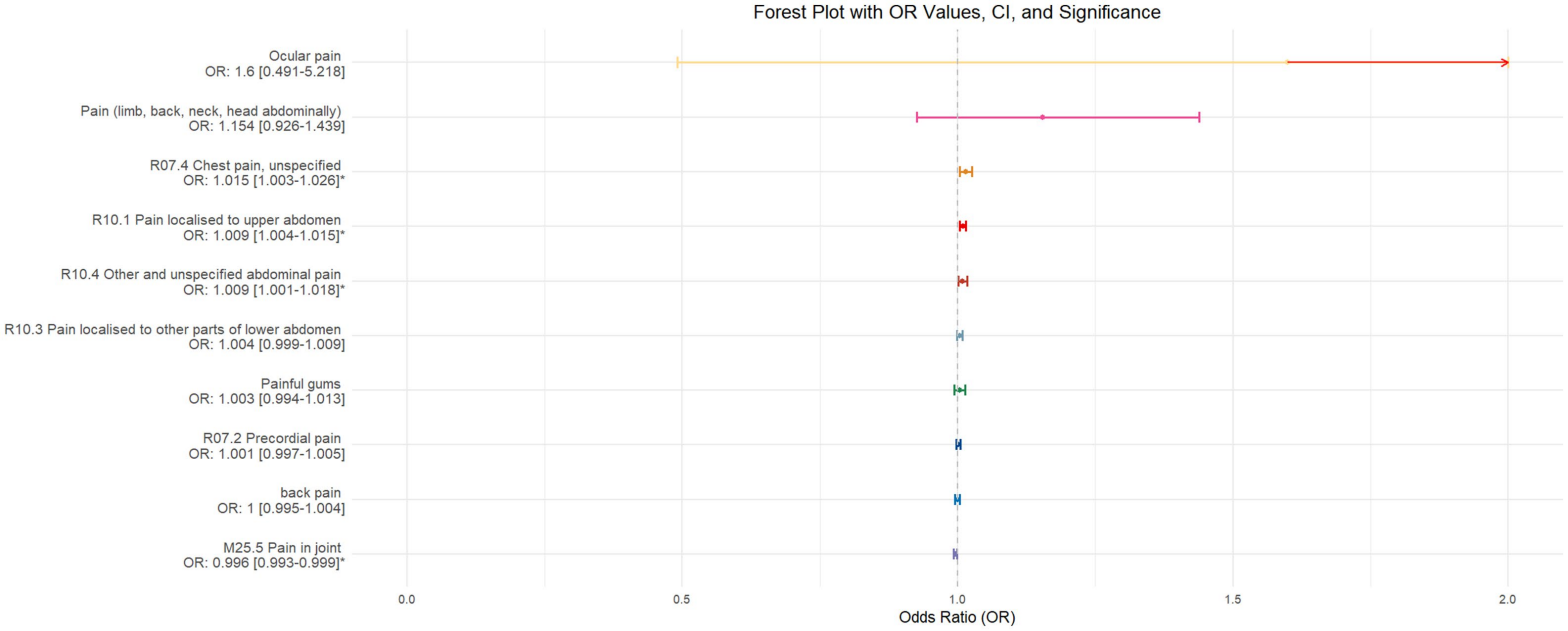
Forest plot of IVW MR analysis results using daytime napping as the exposure factor and pain perception in 10 different body regions as outcomes, ordered by OR value from top to bottom. * Indicates statistically significant MR analysis results by IVW method. In this context, a leftward arrow indicates that both the OR value and the upper limit of the OR are not fully displayed, whereas a rightward arrow signifies that only the upper limit of the OR is not fully displayed.

**Figure 8:**
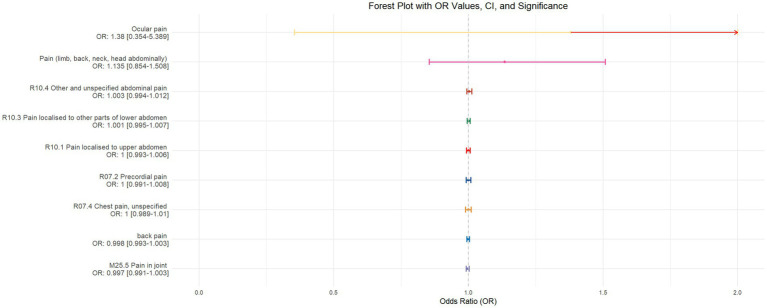
Forest plot of IVW MR analysis results using chronotype as the exposure factor and pain perception in 10 different body regions as outcomes, ordered by OR value from top to bottom. * Indicates statistically significant MR analysis results by IVW method. In this context, a leftward arrow indicates that both the OR value and the upper limit of the OR are not fully displayed, whereas a rightward arrow signifies that only the upper limit of the OR is not fully displayed.

**Figure 9:**
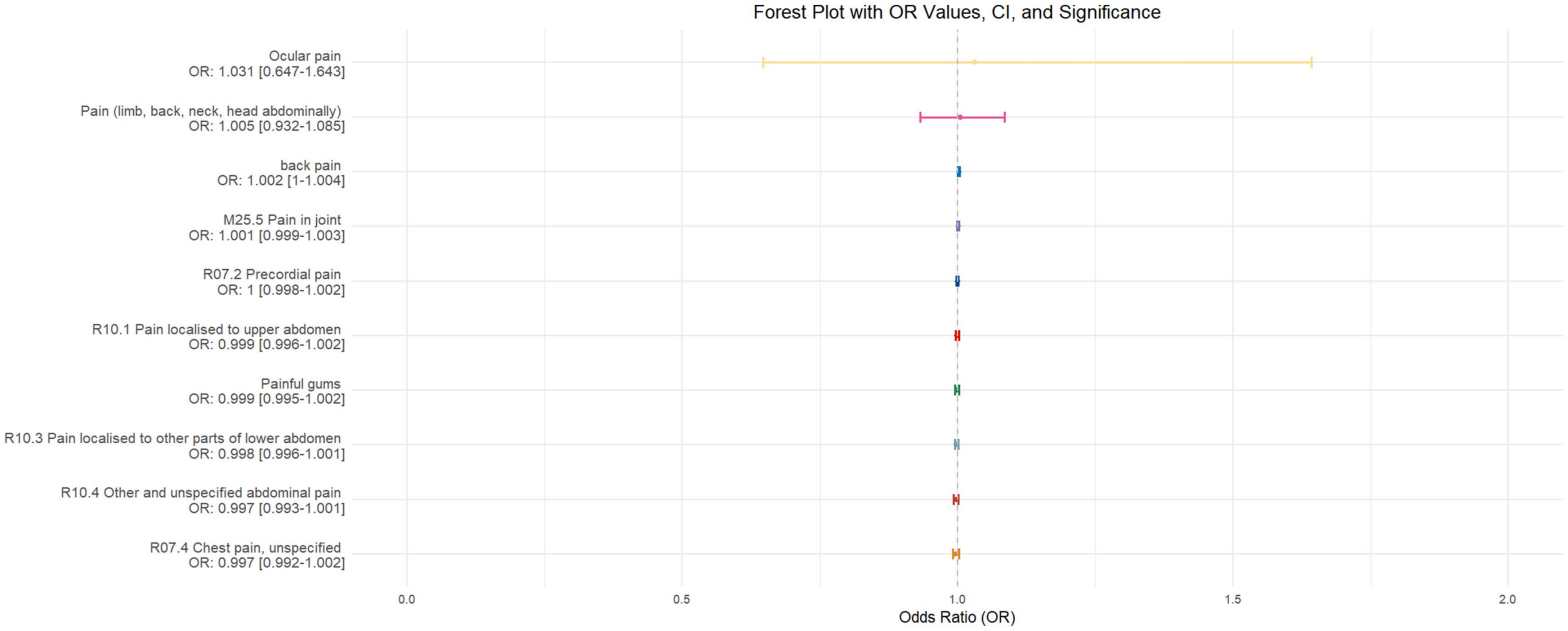
Forest plot depicting the IVW MR analysis results with sleep duration as the exposure factor and pain perception across 10 different body regions as outcomes, ordered by odds ratio (OR) values from top to bottom. An asterisk (*) denotes statistically significant MR analysis results using the IVW method. In this context, a leftward arrow indicates that both the OR value and the upper limit of the OR are not fully displayed, whereas a rightward arrow signifies that only the upper limit of the OR is not fully displayed.

The validation of MR analysis confirms that insomnia increases the risk of unspecified pain, chest pain, gum pain, upper abdominal pain, and lower abdominal pain, as shown in Figure 6. Conversely, daytime napping may elevate the risk of chest pain, upper abdominal pain, and generalized abdominal pain while potentially reducing the risk of joint pain, as illustrated in Figure 7. Neither sleep chronotype nor sleep duration exhibited a significant causal relationship with pain perception, as depicted in Figures 8, 9.

## 4 Discussion

This two-sample Mendelian randomization study investigated the causal relationships between four sleep characteristics (sleep duration, insomnia, circadian rhythm, and daytime napping) and pain in 10 different locations (including limbs, back, neck, head, abdomen), eye pain, chest pain, precordial pain, generalized abdominal pain, upper abdominal pain, lower abdominal pain, gum pain, joint pain, and back pain. The results indicate a certain causal relationship between sleep characteristics and the sensation of pain in various body parts. Overall, insomnia/sleep deprivation appears to contribute more significantly to pain in multiple body parts, whereas sufficient sleep is associated with a relatively lower probability of bodily pain and contributes less significantly.

There are certain discrepancies between the exploratory and validation results of this study. However, the validation MR analysis appears to correct the biases present in the exploratory MR analysis, rendering the study conclusions more conservative and robust. The causal relationship between insomnia and back pain remains inconclusive. In the preliminary exploration, insomnia increased the likelihood of back pain by 1.387 times, whereas this association did not attain significance in the validation MR analysis. Analyzing the data sources, the potential reason for this discrepancy could be that in the initial MR analysis, insomnia data were sourced from the UK Biobank, while back pain data were derived from the EBI database. This configuration minimized the likelihood of sample overlap, possibly yielding more scientifically accurate MR analysis results. In the validation MR analysis, in an effort to minimize the overlap rate between exposure and outcome samples while considering the accessibility of the corresponding phenotypes from the Open GWAS database, insomnia and back pain data were sourced from different batches within the same database. This adjustment may have introduced bias into the study results. To verify the accuracy of our analysis, we reviewed the literature and found supporting evidence. A cross-sectional study conducted by Barazzetti et al. (2022) based on a female population demonstrated that insomnia increases the likelihood of chronic low back pain. Additionally, a study by Roman-Juan et al. (2024) utilizing data from the HBSC study from 2002 to 2018 confirmed that insomnia increases the incidence of back pain in adolescents. These findings from different populations further corroborate the causal relationship between insomnia and back pain, particularly low back pain.

To date, there are relatively few observational studies on the causal relationship between sleep and pain, and the causal link and biological mechanisms between sleep and the sensation of bodily pain remain unclear. A longitudinal cohort study on adults aged 18–25 years (Bonvanie et al., 2016) demonstrated an association between sleep disorders and the severity of chronic pain, musculoskeletal pain, headaches, and abdominal pain, predicting an increase in chronic pain and musculoskeletal pain over the next 3 years. This predictive effect was more pronounced in women than in men, with fatigue serving as a primary mediator rather than anxiety, depression, or reduced physical activity. Moreover, a cross-sectional study based on electronic health record biobanks by Dashti et al. (2021) found a correlation between shorter bed rest time and a higher incidence of acute pain. A meta-analysis by Varallo et al. (2022) also indicated that sleep deprivation enhances pain perception in individuals with chronic pain and that sleep fragmentation increases pain sensitivity in peripheral and central nerves. Additionally, a scientific study conducted by Jiang et al. (2024a) analyzed the association between nocturnal sleep and daytime napping with painful temporomandibular joint disorder (TMD). The results revealed that patients with poor sleep quality (PSQI ≥6) had higher FAI (Fonseca Anamnestic Index) scores (median 60, *p* < 0.001) and a greater prevalence of painful TMD. The myalgia subgroup had higher PSQI (Pittsburgh Sleep Quality Index) scores (median 8, *p* < 0.001) compared to the arthralgia subgroup. Yang Jiang et al.’s research suggested that arthralgia is more sensitive to insomnia. However, this phenomenon was not observed in either the initial study or the validation study of our research, which instead noted gum pain associated with insomnia similar to painful TMD. Furthermore, a cross-sectional study based on the UK Biobank conducted by Jiang et al. (2024b) confirmed the bidirectional relationship between pain and sleep. Participants experiencing widespread pain were significantly associated with unhealthy sleep patterns (OR = 1.18, *p* < 0.001) and other sleep characteristics (*p* < 0.05). The risk of chronic orofacial pain exhibited a nonlinear relationship with sleep duration (*p* = 0.032). Mendelian randomization analysis indicated a causal relationship between prolonged sleep and TMD-related pain (OR = 6.77, *p* = 0.006). This study supplements the scientific evidence for the bidirectional association between sleep and pain, corroborating the findings of the present research. The potential mechanisms underlying the relationship between sleep and pain perception primarily include the following two aspects: first, the correlation between sleep and pain is fundamentally related to dopamine (Bannister et al., 2009). Dopamine receptors are widely distributed in the ascending reticular activating system, and the binding of dopamine to these receptors plays a crucial role in maintaining wakefulness. Correspondingly, dopamine is also a key neurotransmitter in the endogenous pain system. Therefore, when sleep disorders or chronic pain cause dysregulation of dopamine levels in the internal environment, it may lead to corresponding changes in pain perception and sleep characteristics. Second, preclinical studies have shown that sleep deprivation can disrupt the endogenous opioid system and weaken the analgesic efficacy of μ-opioid receptor agonists (Nascimento et al., 2007). The specific mechanism is that sleep deprivation alters the function of opioid receptors in the mesolimbic circuit, particularly the μ and δ receptors, reduces baseline levels of endogenous opioids, and downregulates central opioid receptors. This, in turn, diminishes analgesic efficacy and enhances pain perception. These findings are generally consistent with our study, supporting the conclusion that sleep deprivation contributes to the sensation of bodily pain and, to some extent, validating the related conclusions of our research.

This study has several advantages: (1) It is the first two-sample Mendelian randomization study to explore the causal relationships between four sleep characteristics and pain in 10 different locations, providing reference for research on sleep deprivation leading to bodily pain and the specificity of pain locations; (2) The study population consists of Europeans, representing a demographic with typical sleep deprivation issues, making this research more representative; (3) The use of the MR PRESSO method with 1,000 distributions to remove outliers enhances the reliability of the research findings; (4)This study utilized publicly available data from different databases to validate the research conclusions, thereby eliminating some of the biases resulting from sample overlap. This approach has rendered the final research conclusions more robust.

However, the study also has limitations: (1) The study population is limited to Europeans, limiting the generalizability of the conclusions; (2) It is a unidirectional two-sample Mendelian randomization study, not exploring the impact of different pain locations on sleep characteristics; (3) The lack of investigation into mediators means the study cannot reveal the specific biological mechanisms underlying the exposure-outcome causal relationship; (4) The study reveals the causal relationship on a genetic prediction level, lacking observational cohort studies or randomized controlled trials across a larger population, all age groups, and all ethnicities to validate the conclusions; (5) The study does not encompass all possible pain locations, limiting its ability to fully elucidate the specific composition of unspecified pain perception associated with insomnia.

In conclusion, ensuring sufficient sleep and high sleep quality plays an important role in reducing the sensation of bodily pain. Further validation through larger-scale observational cohorts by researchers in the field is still needed.

## Data Availability

The original contributions presented in the study are included in the article/Supplementary material; further inquiries can be directed to the corresponding author.

## Footnotes

^1^ https://gwas.mrcieu.ac.uk/

^2^ http://www.phenoscanner.medschl.cam.ac.uk/

^3^ https://www.drawio.com/
