# Supplementary figures and images for: The causal relationship between sleep characteristics and multi-site pain perception: a two-sample Mendelian randomization study
Source: Front Neurosci. 2024 Aug 13;18:1428951. doi: 10.3389/fnins.2024.1428951 (PMC11347297; doi:10.3389/fnins.2024.1428951)

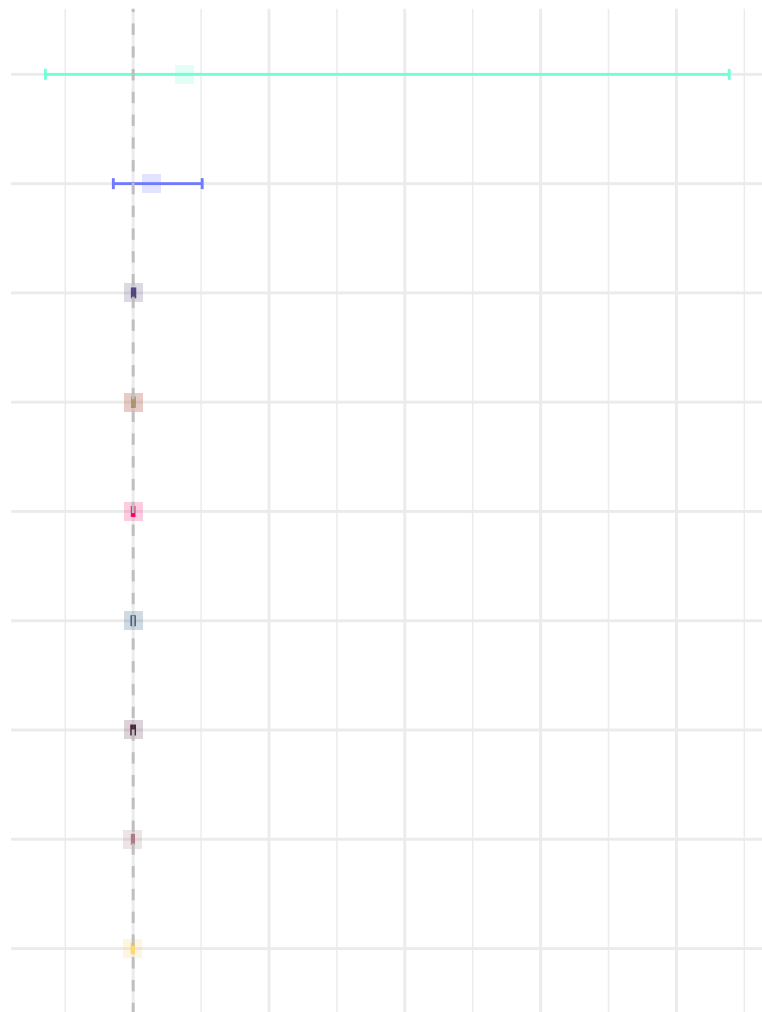

Supplement: Supplementary file 3 [file Data_Sheet_3.ZIP › Figure/New_CT.pdf]

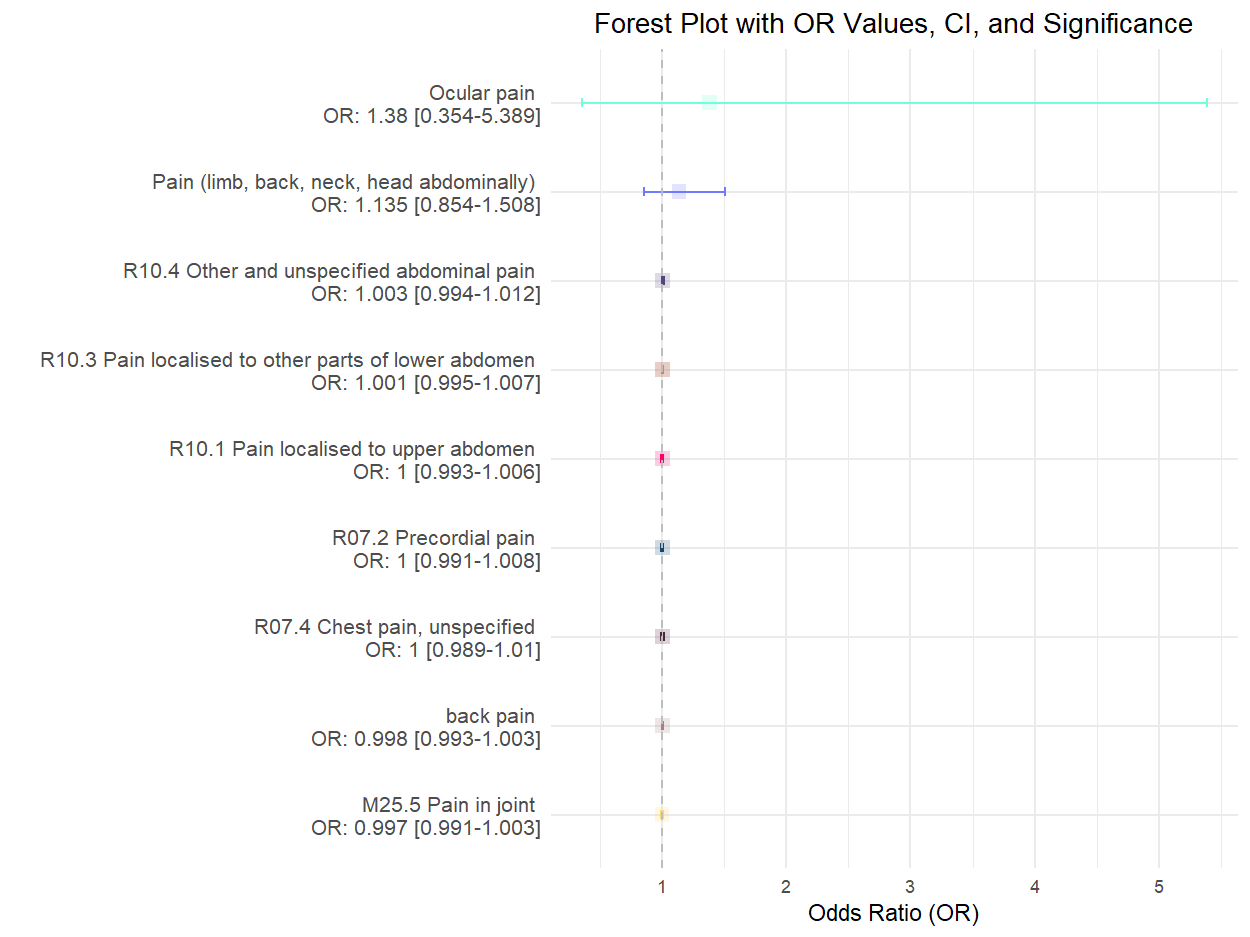

Supplement: Supplementary file 3 [file Data_Sheet_3.ZIP › Figure/New_CT.png]

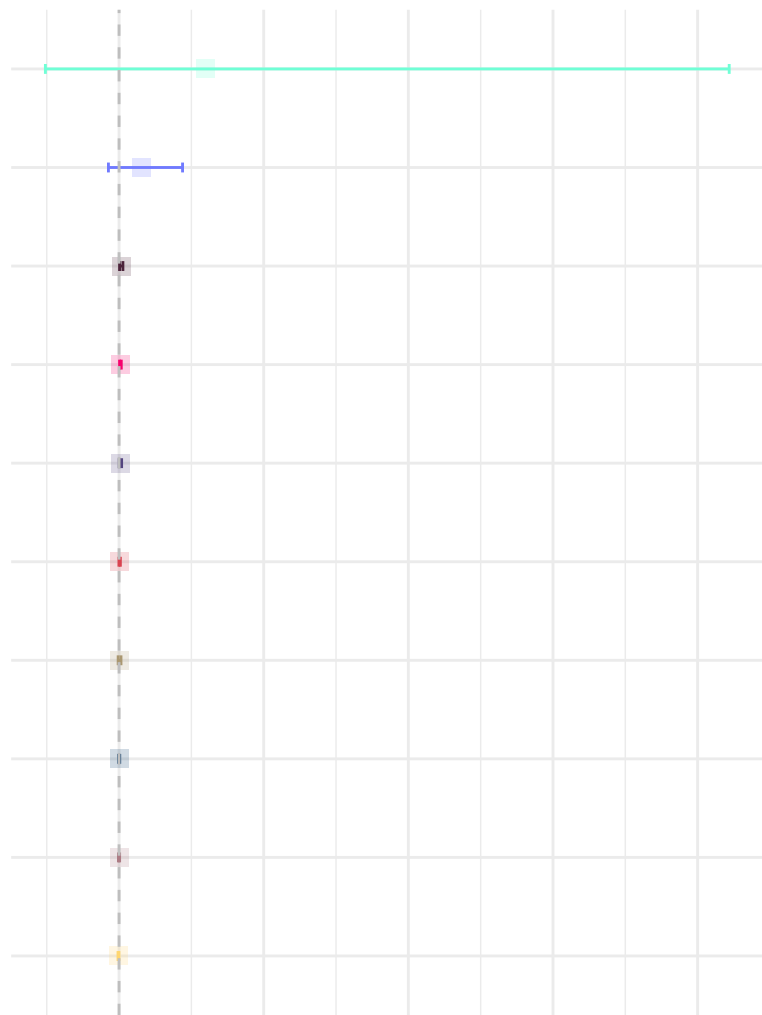

Supplement: Supplementary file 3 [file Data_Sheet_3.ZIP › Figure/New_DN.pdf]

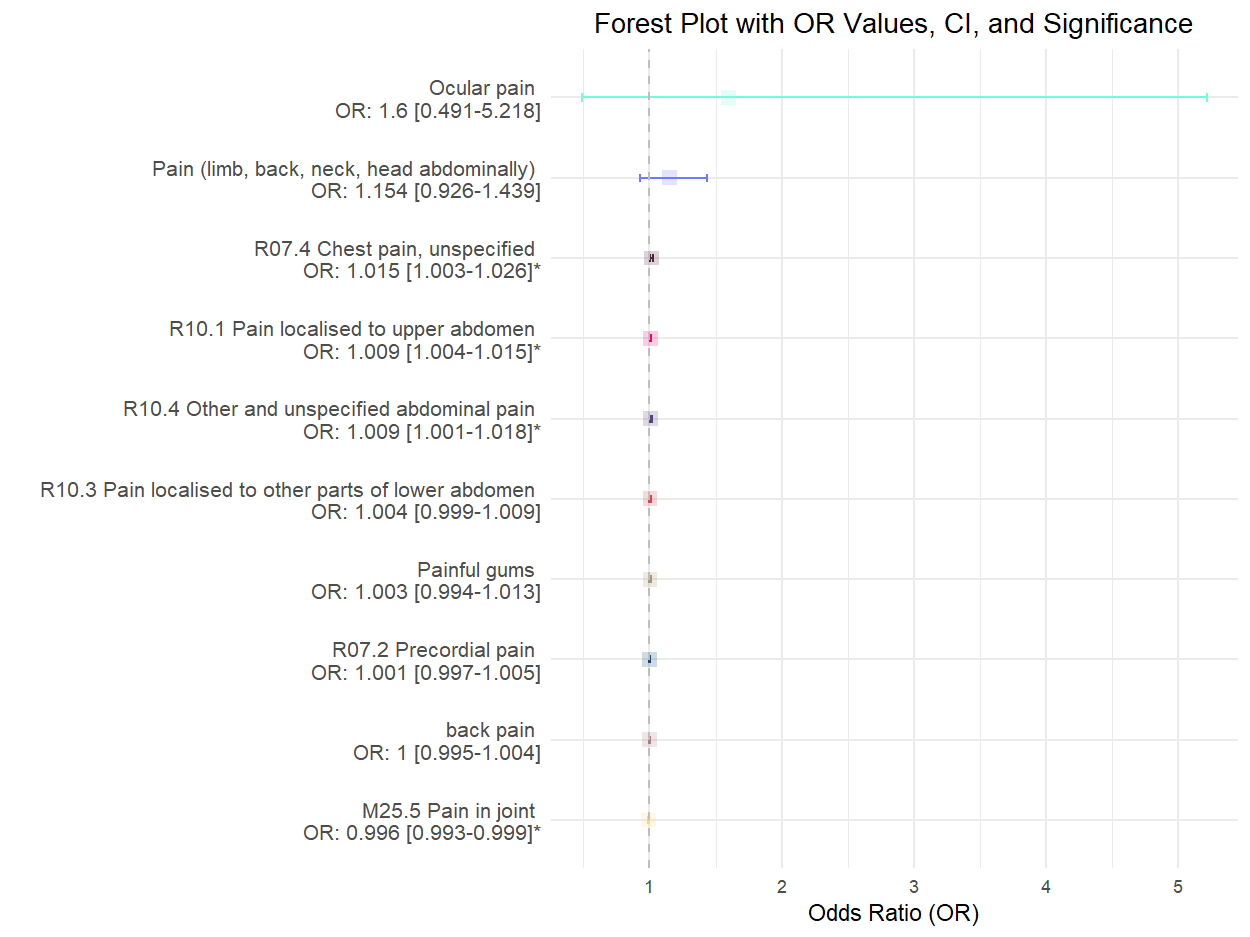

Supplement: Supplementary file 3 [file Data_Sheet_3.ZIP › Figure/New_DN.png]

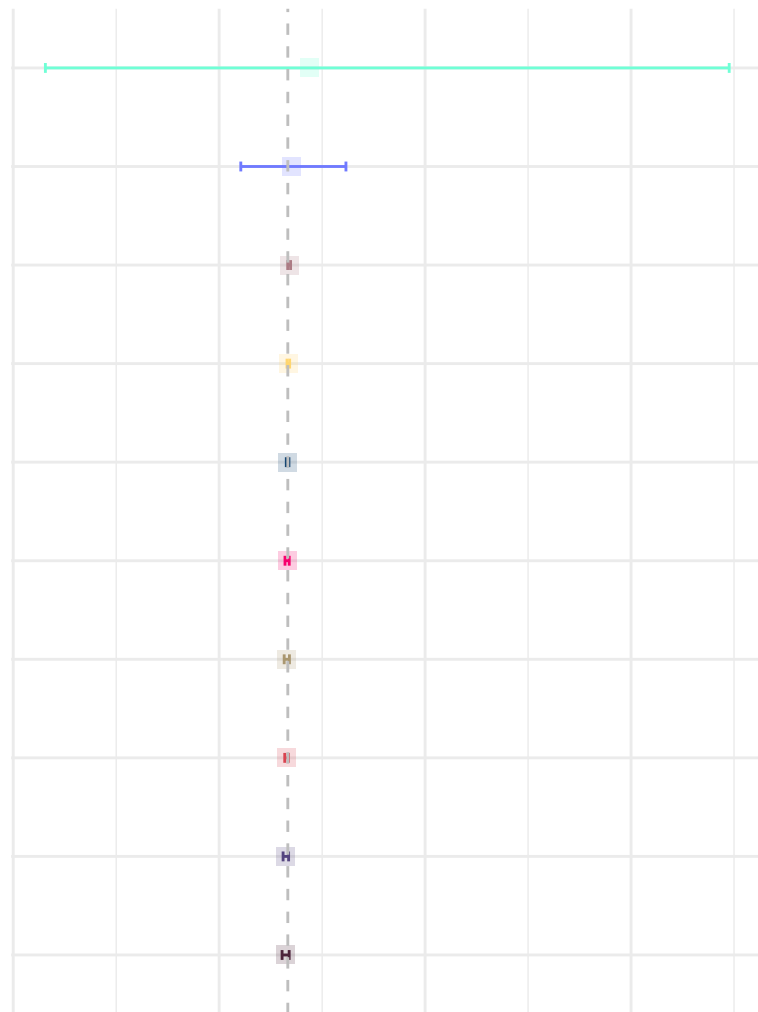

Supplement: Supplementary file 3 [file Data_Sheet_3.ZIP › Figure/New_SD.pdf]

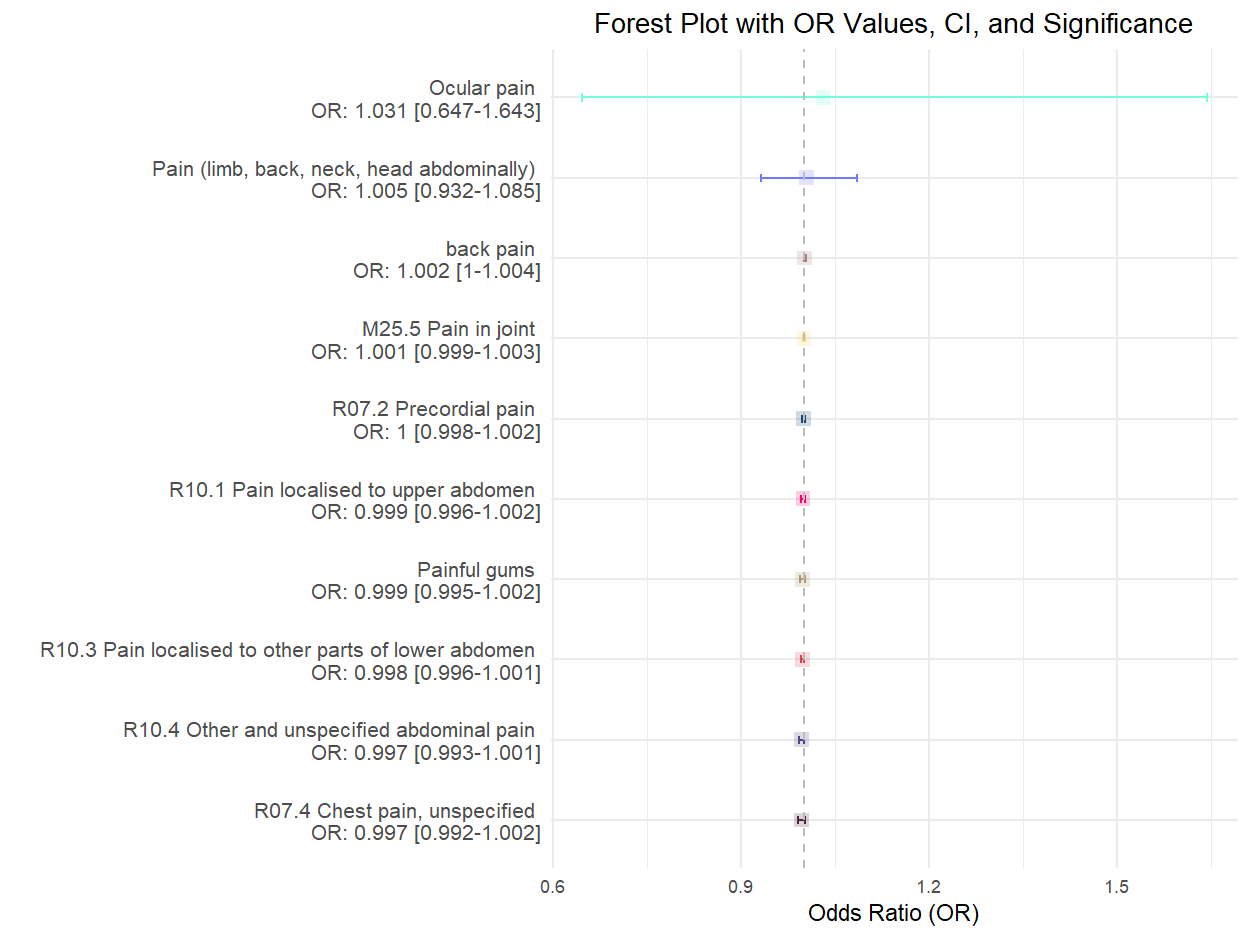

Supplement: Supplementary file 3 [file Data_Sheet_3.ZIP › Figure/New_SD.png]

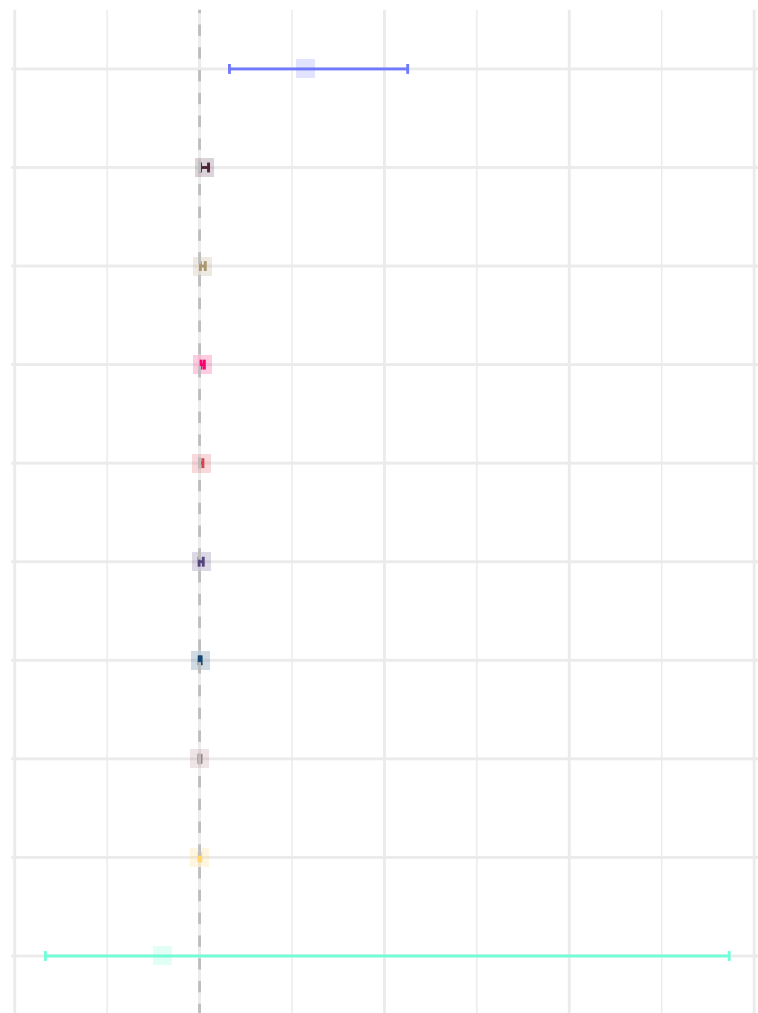

Supplement: Supplementary file 3 [file Data_Sheet_3.ZIP › Figure/New_SL.pdf]

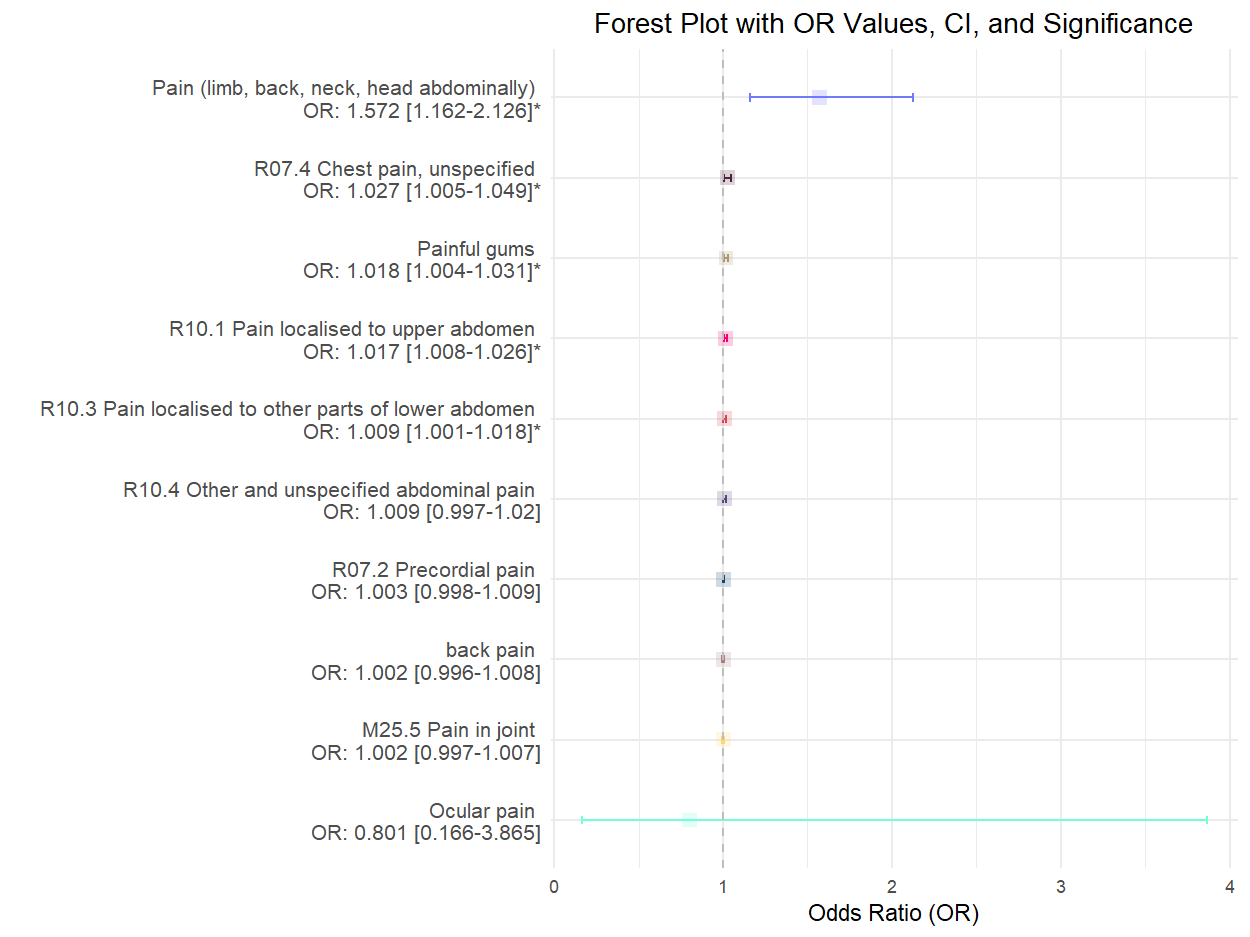

Supplement: Supplementary file 3 [file Data_Sheet_3.ZIP › Figure/New_SL.png]
